# Central Dental Association of Northern New Jersey

**Published:** 1884-08

**Authors:** 


### THE CENTRAL DENTAL ASSOCIATION OF NORTHERN NEW JERSEY.

A regular monthly meeting was held at the residence of Dr. E. H. Bunting, No. 15 New Street, Newark, N. J., May 19th, 1884. After the transaction of the regular routine business, Dr. B. F. Luckey, of Paterson, read a paper upon "The Care and Treatment of Exposed Pulps." (See page 430.)

### DISCUSSION.

*Dr. Watkins*—I would like to explain that Dr. Luckey had not more than two days' notice to prepare this paper, which I consider an exceedingly creditable one.

*Dr. Stockton*—I know that pulps can be fractionally saved ; I am acquainted with a case where part of the pulp was excised thirteen years ago, and the remainder is still alive, and the tooth doing well. Our success depends very much upon the physical condition of the patient. There are those, the pulps of whose teeth becoming only slightly exposed and inflamed, it seems almost impossible to save them ; and there are others with an exposed pulp that has been aching for weeks, and yet it readily yields to treatment. In all doubtful cases it is better to fill the tooth temporarily, for three or six months, and then examine as to the condition before filling permanently. I very rarely fill a tooth at the same sitting at which I have capped the pulp.

*Dr. Cosad*—I am glad it was my privilege, even in my weakness, to be here and listen to the reading of so intelligent a paper.

*Dr. Meeker*—I have used a chloroform solution of gutta-percha for a number of years as a pulp covering, but I think it is liable to swell when used in a pasty condition directly over the exposure. I have met with gratifying success in the use of iodoform paste and asbestos.

*Dr. Watkins*—I have used asbestos felt for the past two weeks, in perhaps ten or twelve cases, for capping pulps where there was slight exposure, and where the pulp was almost exposed. In regard to its working qualities, my experience has been that it works beautifully under the instrument. It is soft and easily placed in position, and does not adhere to the instrument, or draw away like gutta-percha or other materials used in a similar way. After I have the cavity all prepared I cover the pulp with iodoform, cut a piece of felt the size the case requires, place it upon the instrument, touch it upon some liquid gutta-percha, and carry it carefully over the pulp, and now you will see the beauty of the felt. On account of its soft working qualities you can tap it all around the edges, and bring it closely down to the dentine, and there is no moving or shifting from place. You all know that I use iodoform, and how much I rely upon it in almost all cases of exposed pulps, or where the pulp is decomposed in preparing pulp canals, etc. I have had two cases in which I have used iodoform, that I would like to speak of. The first was a child about four years of age—left superior first temporary molar, posterior approximal surface badly decayed. In excavating, the pulp was slightly exposed, which I did not discover at the time I filled the tooth with gutta-percha. The child returned in about two days and complained that the tooth was aching. Upon removing the filling a small drop of pus exuded, which was the first information I had of the exposure. I washed it out with warm water, then with creosote, capped it, and again filled with gutta-percha. The child went home perfectly easy, but at the end of two days I had to repeat the same thing, and after trying different remedies several times with the same result, I decided to give iodoform a trial, for just about that time I heard Dr. Bodecker read a paper before the Odontological Society, where he gave a prescription to prepare the paste, and a description of its use and success in his hands. The next time the child came in I placed some of the paste over the pulp, and covered it with a saucer-shaped cap of hard rubber, made very thin and filled with gutta-percha, and that is the last I have ever seen or heard from that tooth.

The second case was that of a boy twelve years of age, who broke the right superior central incisor diagonally across the center, exposing the pulp freely. I covered it with iodoform, and ran liquid gutta-percha (thick) over that, then filled with oxy-phosphate.

It has been my pleasure to see each of these cases during the past week. The first one had the appearance of a thin film of bone forming over the pulp, but on account of its position I dare not touch it to make sure, but refilled it with oxy-phosphate. In the second case there was a decided formation of secondary dentine over the pulp, and no sign of any exposure, so I built it down with gold, and do not expect to hear from it again. So much for iodoform. I have not so much faith as some of you have in saving pulps where there is much inflammation. Of late I have rarely tried to save them under these circumstances. Of course, I cap where there is not much inflammation, and make great effort to save them, but you know much depends upon the condition of the patient.

I have had many cases of dead pulps come into my hands for treatment after they had been capped, and some of them by our most eminent dentists, who were anxious to save them alive. I think, however, that more pulps can be saved alive with iodoform than with any other remedy. As for amputating pulps, I do not believe in it at all, and I think if those who are over-anxious to save parts of pulps alive would open them two years afterward, they would find a high state of putrefaction. I have found this to be so in all cases which it has been my lot to examine.

*Dr. Luckey*—I do not know whether the preparation of iodoform paste that I use is better than that recommended by Dr. Bodecker or not, but I *do* know that my treatment is successful, and is very seldom followed by any adverse results. Dr. Watkins believes that it is not possible to amputate and save a portion of the pulp alive, but that all such attempts are unsuccessful, and that the remaining portion becomes a putrefying mass. I have treated a number of cases where I removed fully one-half of the pulp, and those teeth to-day are comfortable and doing good service, with all the indications and appearances of healthy normal teeth. I have not been inquisitive enough to open any such teeth for the purpose of ascertaining whether they were alive or not, but I think from their appearance and behavior that it is safe to assume that they are alive, for if such was not the case we all know from experience what trouble would be likely to result.

*Dr. Watkins*—I believe it is owing to the presence of iodoform that putrefaction is prevented, and as long as this is secured it is not at all probable that there will be any pain ; but I doubt very much if the pulp is alive in such cases.

*Dr. Meeker*—In capping pulps we often find that considerable inflammation ensues. In that case I use capsicum bags, and find that they do the work effectually.

*Dr. Merritt*—I have used split raisins sprinkled with cayenne, and find them as effectual as the capsicum bags.

